# *ATXN2* a culprit with multiple facets

**DOI:** 10.18632/oncotarget.17112

**Published:** 2017-04-14

**Authors:** Aurore Nkiliza, Marie-Christine Chartier-Harlin

**Affiliations:** Université de Lille, Inserm, CHU Lille, UMR-S1172, JPArc, Centre de Recherche Jean-Pierre AUBERT Neurosciences et Cancer, and Inserm, UMR-S 1172, Team “Early stages of Parkinson's disease”, Lille, France

**Keywords:** αtaxin-2, CAG repeats, polyA RNA binding, RNA metabolism, Parkinson's disease

*ATXN2* encodes Ataxin-2, a protein involved in RNA metabolism and metabolic homeostasis [1]. CAG repeat expansions in this gene are mostly known for their role in Spinocerebellar Ataxia type 2 (herein referred to as SCA2c) but proofs of their implication as a risk factor of amyotrophic lateral sclerosis (ALS) increase constantly. Growing evidences have been reported showing that these mutations could be associated with Parkinson's disease (PD), but a meta-analysis failed to reveal significant association even though evidence of linkage to CAG repeats at the *ATXN2* locus was found for familial PD [2, 3].

To seek the complex role of CAG repeats in familial PD (herein referred to as SCA2p), we performed a without a priori transcriptome analysis of mononuclear blood cells from SCA2c, SCA2p as well as sporadic PD without *ATXN2* mutations and controls [4]. We found a specific pattern of deregulated pathways linked to cellular proliferation and differentiation associated with the SCA2p group. These data suggest that these processes are important in the development of the PD phenotype and could be one of the consequences associated with *ATXN2* triplet expansions. Notably, we observed strong similarities between sporadic PD and the SCA2p patients with involvement of molecular pathways already described in PD. Of interest, we also observed gene expression perturbations of the ALS signaling in SCA2p and sporadic PD compared to controls. Disturbances of this pathway also showed up after our analysis of Yokoshi and collaborator's data from high-throughput sequencing of Ataxin-2-bound RNAs [5]. They reported that Ataxin-2 could bind RNAs in a dependent and independent manner on PABPC1, a translation factor being a member of PABP family. Analyzing Ataxin-2 RNAs targets, we revealed that some of these Ataxin-2 targets requiring Ataxin-2/PABPC1 interaction to be recognized belong to ALS signaling. Moreover, we and others reported down-regulations of several members of PABP family in PD, more especially PABPC1 and PABPC4. The discovery of the link between ALS signaling and both PD phenotype and Ataxin-2/PABPC1 raises the question of the role of PABP proteins in PD. Of note, we also observed that deregulated genes of ALS signaling included heterogeneous nuclear ribonucleoprotein (hnRNP). Knowing that hnRNPA1/B2, hnRNPA1 and hnRNPA3 are known as rare causes of ALS and that a decrease of hnRNPA1 is associated to aggregation of TDP-43 in ALS, it would be interesting to further explore hnRNP family members in patients with a PD phenotype [4].

The possible mechanisms contributing to neurodegeneration are multiple, with neuronal toxicity being one of the first associated with CAG repeat expansions. RNA metabolism dysfunctions including the sequestration of RNA binding proteins, have emerged as a deleterious feature of several neurodegenerative disorders. Given that Ataxin-2 is involved in RNA metabolism, we could expect deregulations of this fundamental mechanism induced by CAG expansions. Indeed, in SCA2p, sporadic PD but also SCA2c group, our transcriptome analysis revealed a high disturbance of molecular functions linked to RNA binding, polyA RNA binding, mRNA binding as well as structural constituent of ribosome. We found numerous functions linked to translation in patients with a PD phenotype (SCA2p and sporadic PD) compared to SCA2c. RNA transport and mRNA surveillance pathways were perturbed in SCA2c, while splicing deregulations were found in sporadic PD and SCA2c. These data show that RNA metabolism disturbances are a common feature of these different forms of degenerative diseases suggesting the involvement of potential similar biological processes but also specificities for each type of patients. Thus, some of the deleterious mechanism might be similar between these 2 disorders. Of interest a link between *SNCA* overexpression and several proteins regulating translation including *ATXN2* as well as a decrease in translation initiation factors has recently been identified [6], comforting a role of translation in PD which can be due in part to Ataxin-2 [7]. Moreover, a new mechanism of toxicity linked to expansions in *ATXN2* [8] was revealed by bidirectional expression of *ATXN2* resulting in the synthesis of a toxic anti-sense *ATXN2* transcript with CUG repeats in SCA2c and ALS patient samples. It is thus possible that such mechanisms also contribute to the physiopathology of PD all the more so as we observed a significant decrease of Ataxin-2 expression in Epstein–Barr virus immortalized from SCA2c and SCA2p patients with more than 35 CAG compared to controls.

Taken together, these recent data support our conclusion underlying the perturbation of RNA metabolisms as a common feature in PD and SCA2. These data not only contribute to a better understanding of the mechanisms leading to PD with CAG expansions, but also conceptually to understanding alternative mechanisms leading to the vast majority of PD cases.

Several questions need to be answered:

- Our study was performed on a small number of patients, will the future transcriptome studies confirm or refute our results?
- Will extended PD patient studies confirm or refute the decreased expression of Ataxin-2 associated of SCA2p with CAG repeats >35 in familial PD?
- Will we find a mutated non coding RNA in *ATXN2* of the SCA2p group with CAG repeats >35?
- Do PABP proteins play a role in physiopathology of PD?
- Since hnRNPs family is involved in TDP-43 aggregation, is it also linked to alpha-synuclein aggregation?
- Since PABPC1 is sequestrated by CAG42 expansions, is it still sequestrated by Ataxin-2 with a lower number of repeats? And/or by aggregated alpha-synuclein?
- Will new PD genes be involved in RNA metabolism, splicing, translation or non-coding RNA?
